# Evaluation of the bone morphology around four types of porous metal implants placed in distal femur of ovariectomized rats

**DOI:** 10.1186/s13018-020-01822-3

**Published:** 2020-08-03

**Authors:** Stanislav Bondarenko, Nataliya Ashukina, Valentyna Maltseva, Gennadiy Ivanov, Ahmed Amine Badnaoui, Ran Schwarzkopf

**Affiliations:** 1 Dept of Joint Pathology, Sytenko Institute of Spine and Joint Pathology, National Academy of Medical Sciences of Ukraine, 80 Pushkinskaya St, Kharkiv, 61024 Ukraine; 2 NYU Langone Medical Center, Hospital for Joint Diseases, New York, USA

**Keywords:** Tantalum, Titanium, Osteoporosis, Animal model, Bone remodeling, Histology

## Abstract

**Background:**

To compare structural features of the femoral bone of ovariectomized and non-ovariectomized rats after implantation of porous materials (TANTALUM, CONCELOC, TTM, ATLANT).

**Methods:**

Experiments were carried out on 56 white laboratory female rats aged 6 months. Rats were randomly assigned into groups: sham-operated control group (SH) or ovariectomy group (OVX). Four different commercial implant materials (TTM, CONCELOC, TANTALUM, ATLANT) were placed into the defects (diameter 2.5 mm, depth 3.0 mm) in the distal metaphysis of femurs. Rats were sacrificed 45 days after surgery. Histological study was performed and the percentage of the bone area (BA%) around the implant at a distance of 500 μm in the cancellous area was measured.

**Results:**

Formation of mature bone tissue of varying degrees around all of the implants was detected. In OVX rats cancellous bone defect zone was characterized by a high density of osteocytes on the surface. In the SH group, no differences in BA% among implant materials were found. In OVX rats, the BA% around ATLANT implants was 1.5-time less (*p* = 0.002) than around TANTALUM. The BA% around the rest of the materials was not statistically different.

**Conclusions:**

Bone formation around the studied porous titanium and tantalum materials in the osteoporosis model was lower than in normal bone. There were differences in bone formation around the different materials in the osteoporosis model, while in the normal bone model, these differences were absent.

## Background

Total hip arthroplasty (THA) in cases of large acetabular defects and in patients with osteoporotic bone can be difficult. Furthermore, achieving a long-term stable fixation of the acetabular cup is challenging [1–4].

Cement usage for acetabular cup fixation in primary THA has fallen out of favor in recent decades mostly due to increased risk of aseptic loosening in mid and long-term follow-up [5–8]. Biological fixation of highly porous biomaterials in THA plays an important role in long-term survivorship of acetabular implants. A long-term stable fixation of these implants depends largely on the osseointegration of bone tissue into them [9].

Following acetabular component placement, osseointegration depends on both the quality of bone tissue and the properties of the implant surface [10–12]. New highly porous biomaterials were developed to enhance osseointegration and survivorship of acetabular reconstruction [13]. Current, highly porous biomaterials for THA are made of tantalum and titanium (partial use alloy Ti_6_Al_4_V) [14]. The advantages of porous tantalum are high thermal conductivity and biocompatibility [15, 16], and they have been shown to have good survivorship in long-term follow-up [17, 18]. However, according to the Swedish Hip Arthroplasty Registry and the Australian Orthopaedic Association National Joint Replacement Registry data, Trabecular Metal acetabular components in primary THA showed a higher risk for revision compared with other uncemented acetabular cups [19, 20].

Current research focuses on porous titanium materials characteristics, as well as their comparison with tantalum implants [21, 22]. Titanium porous implants have low thermal conductivity, high yield strength, and low weight and have been shown to have high survival [23] and lower cost compared to tantalum implants [14]. In a 10-year clinical randomized trial of primary THA, porous tantalum monoblock cups exhibited greater stability and 100% survivorship compared to porous-coated titanium monoblock cup [22]. In another study using multivariate logistic regression, the authors compared the survival of porous tantalum and porous titanium acetabular components with primary THA at an average of 44.4 months the authors found no difference in outcomes [21].

Bone changes in osteoporosis may cause additional difficulty in acetabular reconstruction as well as increasing the risk of aseptic failure and loosening. Under these conditions, the requirements for implant materials become even more important. In order to study osseointegration and fixation properties, animal models were established for material evaluation [24]. It has been established that osteoporosis can affect the fixation and osseointegration of implants [25, 26], in particular, giving the different responses of osteoporotic cortical and trabecular bone to material implantation [27]. Therefore, establishing the structural features of bone tissue around porous implant materials is important for selecting a particular material for use in patients with low bone mineral density.

In an early study, we compared the osseointegration of a tantalum material with titanium in an ovariectomy model, revealing the highest rate of osseointegration with the tantalum material, while the overall rates of osseointegration in animals with osteoporosis were lower than non-osteoporotic animals [28]. As a result of this, we decided to study several available titanium materials on the market, as well as one novel material in a similar experiment to understand the formation and osseointegration of bone around these materials in an osteoporosis model in comparison with a normal bone model.

The aim of this study was to compare structural features of the femoral bone in ovariectomized and non-ovariectomized rats after implantation of different porous materials.

## Methods

### Animals

Experiments were carried out on 56 white laboratory female rats aged 6 months, with an average body weight of 300 ± 25 g. Rats were randomly assigned to 1 of 2 groups: sham-operated control group (SH) or ovariectomy group (OVX). The study followed the requirements of the European Convention for the protection of vertebrate animals used for experimental and other scientific purposes. The study design was approved by the institution Bioethics Committee (Protocol No. 175 dated 26 February 2018).

### Implants

Four commercially available porous implants were used in this study. The first is TANTALUM—porous Tantalum Trabecular Metal (Zimmer, Warsaw, IN, USA). The other three were made from titanium alloy Ti_6_Al_4_V using additive technologies: TTM (AK Medical, Beijing, China), CONCELOC (Smith & Nephew, Memphis, TN, USA), and ATLANT (TITAN-MED, Kyiv, Ukraine). All materials were comparable in porosity—80% or upper. TANTALUM has an elastic modulus of 3 GPa which is between that of cortical and cancellous bone, and similar to subchondral bone [29]. The elastic modulus of the other three materials was 12.9 GPa for TTM [30], 4.3 GPa for CONCELOC [31], and 113 GPa for ATLANT.

### Surgical procedures

**Fig. 1:**
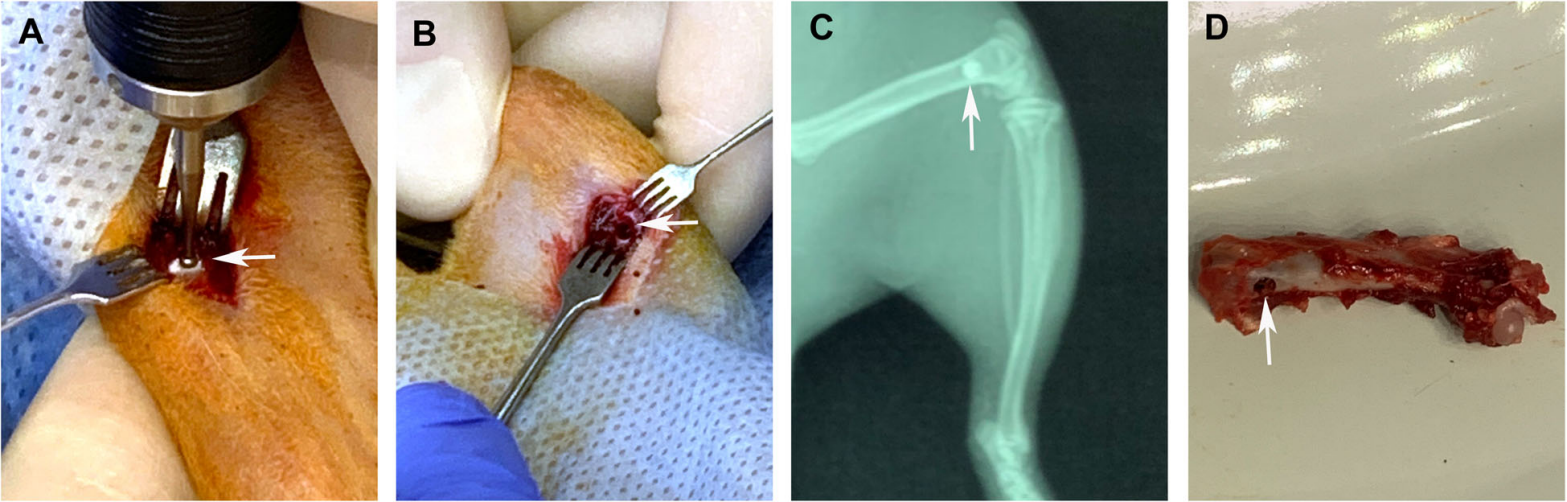
Creation of a defect (arrow) in the distal rat femur (**a**). The created defect (arrow) (**b**). X-ray of the rat femur immediately after implantation with the implant placed (arrow) (**c**). The femur of the rat after euthanasia with an implant in the defect 45 days after implantation (arrow) (**d**)

All surgeries were performed under general intramuscular anesthesia—ketamine 50 mg/kg. Bilateral ovariectomies were performed in 28 animals in the OVX group according to previously described methodology [28]. For the SH group, the ovaries were not removed. At 3 months after ovariectomy, when osteoporotic bone changes had developed in the OVX group [28], surgery was performed in both groups (OVX and SH) of rats. The skin of the lower extremities was shaved and treated with Betadine® solution. From the lateral access, transcortical defects (diameter 2.5 mm, depth 3.0 mm) were created into the distal metaphysis of the left femur using a dental burr (Fig. 1a, b). The implants (TANTALUM, TTM, CONCELOC, or ATLANT) were placed into the prepared defects (Fig. 1c) by using a press-fit technique. Wounds were treated with antibiotic powder and sutured in layers.

The euthanasia of rats was performed 45 days after surgery by administering a lethal dose of anesthetic (sodium thiopental, 90 mg/kg intramuscularly). This observation period was chosen for the reason that other researchers conducted similar experimental studies on rats in the same period [32], as well as the results of the previous study performed by us [28], since for this period (45 days), the site of defect is substituted by new formed mature bone.

### Histological study

After extraction, the implanted left femurs (Fig. 1d) were fixed in a solution of 10% formalin and decalcified in a 10% solution of formic acid. After decalcification, the implants were carefully removed, and the distal metaphysis of the femurs was dehydrated in alcohols of increasing concentration and a mixture of paraffin and xylene (1:1) and embedded into paraffin. Longitudinal histological sections of 5–6 μm (7 of each sample) thick were stained with hematoxylin and eosin (H&E). The bone structure was analyzed under light microscope BX63 (Olympus, Japan) and imaged with a digital camera DP73 (Olympus).

### Histomorphometry

Three sections were obtained from each femoral distal metaphysis. The percentage of peri-implant bone area (BA%) around the implant at a distance of 500 μm in the cancellous area was measured as described previously [26, 33]. The BA% allows us to evaluate all bone areas, not only along the perimeter of the implant, but also at a greater distance from the implant. This is especially important in osteoporosis, when the overall bone area/mass is reduced. The measurements were made using CellSens Dimension 1.8.1 software (Olympus, 2013) for the Olympus BX-63 microscope (at a magnification rate of × 4).

### Statistical analysis

Measurement data were presented as mean ± standard deviation (SD). Comparison of the values in the groups of OVX rats and SH rats was performed by using the one-way ANOVA analysis with Bonferroni post-test. To compare values between OXV and SH groups, we used unpaired *t* test. A critical level of significance was accepted as 0.05. The analysis was performed using the IBM SPSS Statistics 19.0 software.

## Results

During *histological examination* at 45 days after surgery in animals of both groups, a formation of mature bone tissue with varying degrees around all the tested implants was detected.

In SH rats, there were bone trabeculae of lamellar structure with red and yellow bone marrow in the intertrabecular spaces. Their structure did not differ between the different types of materials evaluated.

**Fig. 2:**
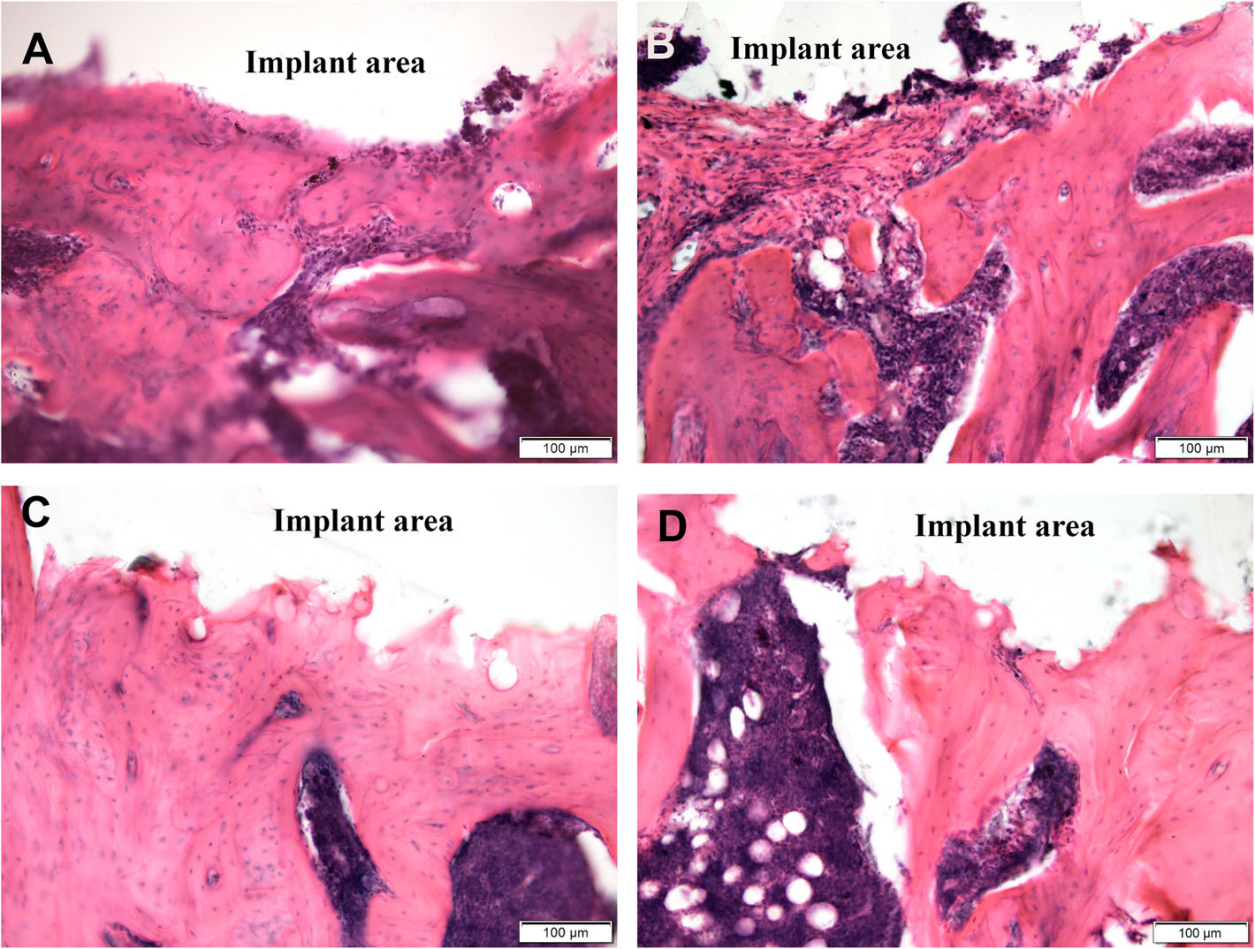
Histological features of newly-formed bone in the cancellous bone defect zone in distal femoral metaphysis of the OVX rats. Forty-five days after implantation of TANTALUM (**a**), TTM (**b**), CONCELOC (**c**), or ATLANT (**d**). H&E

In OVX rats in the cancellous bone defect zone (Fig. 2a–d), newly formed bone trabeculae were characterized by a high density of osteocytes. These bone trabeculae shaped around the implants formed a network. At the same time, the host bone trabeculae were thin, located vertically, at a distance from each other, and did not create the trabecular network. The signs of a reorganization of the newly formed bone were detected in all groups. There were some debris structures of osteons, dilated vascular channels with the formation of connective tissue and bone marrow. In some trabeculae, destructive gaps filled with tissue fluid were observed, as well as areas without cells. In small areas on the surface of the implants, woven bone and connective tissue (Fig. 2b) was located.

**Fig. 3:**
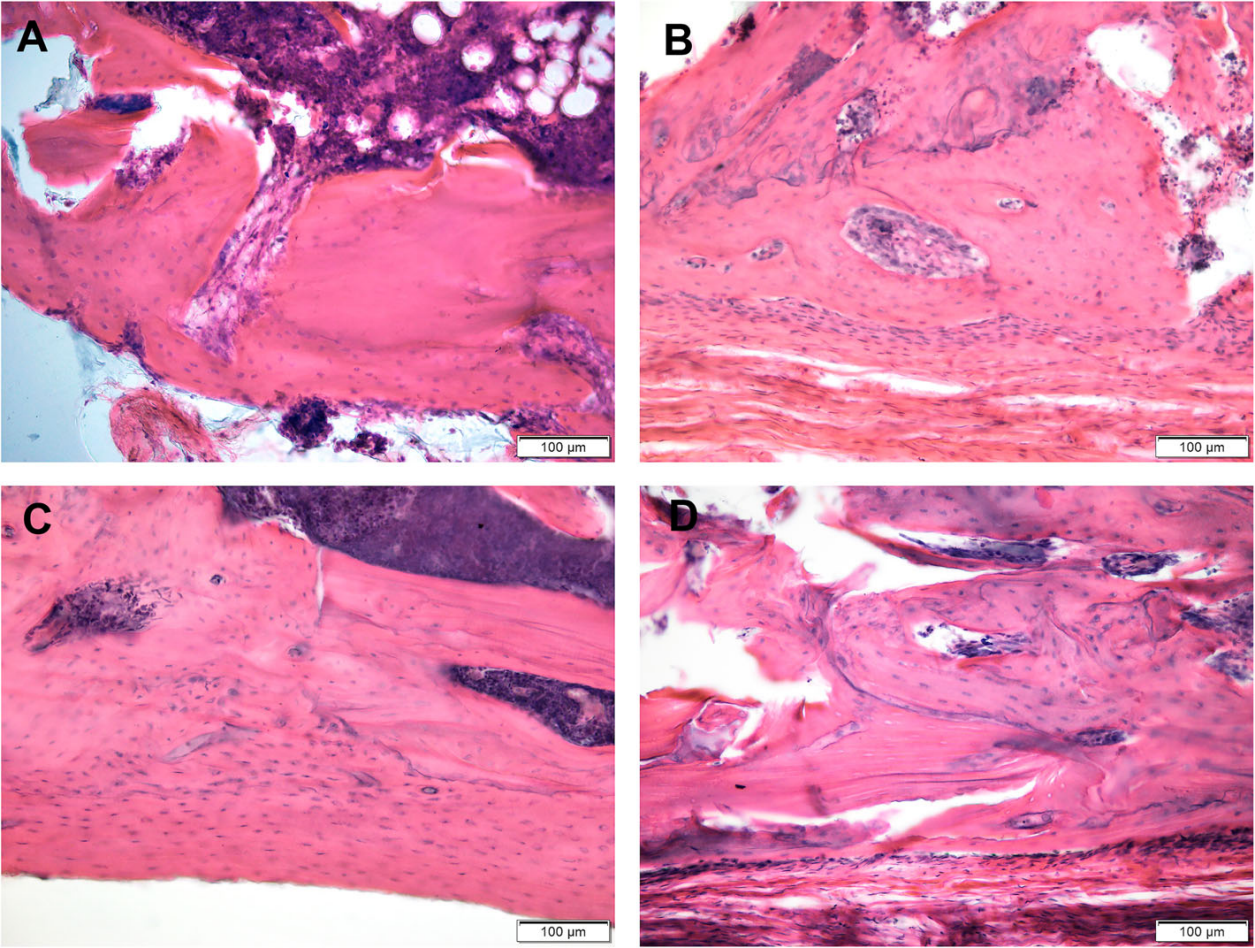
Remodeling of the new-formed bone in the cortex defect zone in distal femoral metaphysis of the OVX rats. Forty-five days after implantation of TANTALUM (**a**), TTM (**b**), CONCELOC (**c**), or ATLANT (**d**). H&E

In the cortex defect (Fig. 3a–d) in the OVX group, trabecular bone was observed as well. This trabecular bone was found to be remodeling and usually strongly connected with the host compact bone and forming periosteal regenerate. In the host cortex in animals of all groups, especially the section distal from the injury area, destructive changes in the form of a rarefaction and thinning were noted. As a result, structural bone was replaced by cancellous bone.

As a result of *histomorphometry* in the SH group, no differences in BA% among the different implanted materials were found (Table 1).

**Table 1:** BA% around different implants implanted into distal femur metaphysis in sham-ovariectomized and ovariectomized rats (mean ± SD)

The material	Group of rats		Unpaired *t* test
	Sham-ovariectomized	Ovariectomized	
TANTALUM	21.09 ± 7.96	10.89 ± 2.47	*p* < 0.0001
TTM	18.76 ± 5.97	9.67 ± 4.03	*p* < 0.0001
CONCELOC	22.67 ± 6.88	7.99 ± 3.37	*p* < 0.0001
ATLANT	19.87 ± 4.92	7.37 ± 2.40*	*p* < 0.0001
ANOVA	p = 0.158	*p* = 0.002	

*p < 0.01 vs TANTALUM in ovariectomized rats’ group (Bonferroni post-test)

In a series of OVX rats, the BA% around ATLANT implants was lower by a factor of 1.5 (*p* = 0.002) compared to TANTALUM. The BA% around CONCELOC and TTM specimens did not differ significantly from indicators in the OVX TANTALUM group.

In the SH group, the BA% around TANTALUM and TTM was higher by a factor of 1.9 (*p* < 0.0001), CONCELOC by a factor of 2.8 (*p* < 0.0001), and ATLANT by a factor of 2.7 (*p* < 0.0001) compared to the OVX group (Table 1).

## Discussion

In our study, carried out in an animal model, we evaluated structural features of the cancellous bone adjacent to four different porous implants: TANTALUM and three others made from titanium alloy Ti_6_Al_4_V using additive technologies—TTM, CONCELOC, and ATLANT. To evaluate their effectiveness in implantation in osteoporotic bone compared to normal bone, we used the generally accepted model of ovariectomy [24]. Our results demonstrated that the type of implant material did not affect the formation and rearrangement of the adjacent cancellous bone after 45 days in animals with normal bone quality (sham ovariectomy group). Similar results were obtained in an experiment on New Zealand rabbits: the authors did not find any differences in osseointegration quality of porous tantalum and porous titanium made by using 3D printing technology at 2, 4, and 8 weeks after implantation in the femoral lateral malleolus [34]. In another study on rabbits, the authors did not find differences in bone growth during implantation of tantalum and titanium porous implants in the femur [35]. In another experimental study on rats, no differences were found in bone formation on the implant surface in the distal femur at the 12th week time point between tantalum and titanium implants [36].

For OVX rats, we set the lowest BA% for ATLANT. In our previous in vivo study, with an osteoporosis model, we observed by way of histomorphometry and histology studies a higher level of osseointegration of trabecular tantalum implants compared to highly porous titanium implants [28]. According to in vitro studies of bone mesenchymal stromal cells of ovariectomized rats, a tantalum substrate exhibits better biocompatibility and osteoinductive properties than titanium [37]. This may contribute to better bone formation around certain implants in cases of osteoporosis. Furthermore, we found lower rates of bone area (BA% decreased by1.9–2.8 times) in OVX rats compared to the SH group. The greatest differences in bone formation were found in OVX rats compared to the SH group with CONCELOC (by 2.8 times) and ATLANT (by 2.7 times). This may confirm the existence of differences in the osseointegration of porous implants with osteoporosis bone. An important role in achieving good long-term results during acetabular reconstruction is influenced by both a correct choice of implant and the bone quality in the periprosthetic acetabular area [10, 38]. It has been shown that in the osteoporotic bone, the rate of migration of uncemented acetabular cups is elevated [2]. Furthermore, periprosthetic fractures may occur [39], which might lead to revision surgery due to decreased fixation of the acetabular cup due to the fragility of the adjacent bone [40]. Recently, a significant amount of porous materials from titanium alloys have been introduced for this purpose [41], an important role in the study of their biomechanical qualities is performed by experimental studies in vivo and in vitro. Current implants made from titanium alloys and tantalum have been successfully used for THA in patients with normal bone mineral density for many years [23]. However, in osteoporotic conditions under changed biomechanical conditions, the long-term survival rate may be reduced [2]. Therefore, the search for the optimal material for hip reconstruction with biomechanical properties resembling the properties of cancellous bone continues.

One of the factors that influenced the results can be Young’s modulus of the materials studied. It has been clinically established that the amount of bone can decrease over a period of 14 years around the femoral stem due to the pressure placed by the implant on the bone, due to the larger Young’s modulus of the implant compared to the bone’s [42]. The use of porous implants from titanium alloy Ti_6_Al_4_V is due to their reduced Young’s modulus, which is closer in value to the cortical bone than to the spongy bone [43]. Experimentally, using finite element algorithms, it was found that a decrease in the stiffness of the material promotes bone growth [44]. The fact that TANTALUM has the lowest Young’s modulus, which is similar in value to the spongy bone among the materials studied, may contribute to the results seen of bone formation (BA%) around such implants in OVX rats. While the lowest BA% in OVX rats was found in the case of implantation of ATLANT material, which had the largest Young’s modulus among the studied materials. Therefore, we assume that the lowest bone formation (BA%) found in the SH and OVX groups for ATLANT is due to the high modulus of elasticity of this material.

Our study is not without limitations; the main limitation of our study was the ability to only measure the bone area around the implants. However, this study shows the peculiarities of bone structure around various materials used in acetabular cup implants in an osteoporotic model in comparison with a normal bone model, which is an understudied topic.

## Conclusions

Bone formation and osseointegration around the studied porous titanium and tantalum materials in the osteoporosis model were lower than in the normal bone model. There are differences in bone formation for various materials in the osteoporosis model, while in the normal bone model, differences were absent.

## Data Availability

Not applicable

## Abbreviations

BA%: Percentage of peri-implant bone area
OVX: Ovariectomy group of rats
SD: Standard deviation
SH: Sham-operated control group of rats
THA: Total hip arthroplasty
